# The Founder: Dispositional Greed, Showbiz, and the Commercial Determinants of Health

**DOI:** 10.3390/ijerph20095616

**Published:** 2023-04-23

**Authors:** Alan C. Logan, Christopher R. D’Adamo, Susan L. Prescott

**Affiliations:** 1 Nova Institute for Health, Baltimore, MD 21231, USA; 2 Department of Family and Community Medicine, University of Maryland School of Medicine, Baltimore, MD 21201, USA; 3 Medical School, University of Western Australia, Nedlands, WA 6009, Australia; 4 The ORIGINS Project, Telethon Kids Institute, Nedlands, WA 6009, Australia

**Keywords:** commercial determinants of health, ultra-processed foods, non-communicable diseases, corporate greed, dispositional greed, psychological traits, collective narcissism, social dominance orientation, planetary health

## Abstract

Marketing unhealthy products by multinational corporations has caused considerable harm to individual health, collective wellbeing, and environmental sustainability. This is a growing threat to all societies and a significant contributor to the rising global burden of non-communicable diseases and early mortality. While there is growing consideration of the commercial determinants of health, this is largely focused on the methods by which unhealthy products are marketed and disseminated, including efforts to manipulate policy. Little attention has been paid to the underlying psychological traits and worldviews that are driving corporate greed. Here, we consider the role of “dispositional greed” in the commercial determinants of health with a focus on the historical attitudes and culture in the ultra-processed food industry—exemplified by “The Founder” of the McDonald’s franchise. We argue that greed and associated psychological constructs, such as social dominance orientation and collective narcissism, permeate the commercial determinants of health at a collective level. This includes how a culture of greed within organizations, and individual dispositional greed, can magnify and cluster at scale, perpetuated by social dominance orientation. We also consider the ways in which “showbiz” marketing specifically targets marginalized populations and vulnerable groups, including children—in ways that are justified, or even celebrated despite clear links to non-communicable diseases and increased mortality. Finally, we consider how greed and exploitative mindsets mirror cultural values and priorities, with trends for increasing collective narcissism at scale, recognizing that many of these attitudes are cultivated in early life. A healthier future will depend on navigating a path that balances material prosperity with physical and spiritual wellbeing. This will require cultural change that places higher value on kindness, reciprocity, and mutualistic values especially in early life, for more equitable flourishing.

## 1. Introduction

“*I expect money like you walk into a room and turn on a light switch or a faucet, it is not enough.*”

—Ray A. Kroc, 1973 [1].

So said the chairman of McDonald’s when *Time* magazine asked how he felt about his-then USD 500 million personal fortune—riches built off his vast and growing empire of fast-food outlets. The quote speaks to dispositional greed and, by extension, corporate greed. Kroc, who referred to himself as founder of the McDonald’s franchise [2,3], went on to tell *Time* that he regarded the fast-food business as showbiz: “*When you are in this business you are in show business. It’s like a Broadway musical—if people come out humming the tune, then the show was a success*”. This speaks to the carnival colors of marketing that underpins the sale of calorie-dense, nutrient-poor ultra-processed foods, and unhealthy products in general. Essentially, a breezy P.T. Barnum-like justification for the theatric persuasion and consumer psychology leveraged to feed Kroc’s desire for money. Kroc offhandedly dismissed concerns about any adverse health effects of his products: “*Mostly, criticism concerns nutrition. My answer is that we sell meat, potatoes, bread, and milk*” [4].

These comments join many others from Kroc—sentiments that, as we will explain below, are at the stone-cold heart of the emerging concept of the “commercial determinants of health”. This narrative review begins with a brief overview of this concept, with a focus on ultra-processed foods and mental health. We contend that psychology is a neglected dimension of existing frameworks of commercial determinants of health. From this standpoint, the authors examine the construct of dispositional greed. We argue that greed and associated psychological constructs, such as social dominance orientation and collective narcissism, permeate the commercial determinants of health at a collective level.

We use Kroc’s fast-food chain, McDonald’s, as not just an important historical example, but as a key part of the foundational mindsets that have shaped corporate attitudes and behaviors. These historical references are not presented here as mere trivia. Rather, we argue that Kroc’s perspectives continue to reverberate through the entire unhealthy product landscape. While McDonald’s is not alone in this, the corporation remains the world’s largest fast-food chain [5] and advertiser of fast foods [6]. Once understood through this lens, it is apparent how the “showbiz” underpinning unhealthy products—that in effect promotes non-communicable diseases and increased mortality—has been justified, or even celebrated.

## 2. The Commercial Determinants of Health

In a 2012 *British Medical Journal* article subtitled “The marketing campaigns of multinational corporations are harming our physical, mental, and collective wellbeing”, Dr. Gerard Hastings argued that the concept of “social determinants of health” needs to expand and consider the “commercial determinants” of ill health with equal vigor [7]. The following year, Dr. Teresa Marteau and Dr. Robert West defined the “commercial determinants of health” as the “factors that influence health which stem from the profit motive” and noted that “the greatest challenge to improving health may lie in the tension between wealth- and health-creation” [8]. This concept was popularized by the extensive writings of political scientist Dr. Ilona Kickbusch [9,10,11,12]. Writing on behalf of the World Health Organization in 2013, Kickbusch and colleague Callum Brindley emphasized that “there is [currently] no discussion of how the commercial determinants of health such as advertising influence the dietary decisions of adults and children. This is a significant omission in light of numerous studies showing this link and the alarming growth in the global disease burden and prevalence of overconsumption” [13].

The term “commercial determinants of health” has slowly entered the academic lexicon over the last decade, with accelerated pace in the last few years. Mostly, this has been in reference to industries hawking potentially unhealthy products—alcohol, ultra-processed foods and beverages (including fast foods), tobacco, firearms, and to a lesser extent, the pharmaceutical, gambling, and mining industries [14]. One indicator that the term has “arrived” is the authoritative academic 2023 textbook entitled *The Commercial Determinants of Health*, from Oxford University Press [15].

The World Health Organization currently defines the commercial determinants of health as “the conditions, actions and omissions by corporate actors that affect health. Commercial determinants arise in the context of the provision of goods or services for payment and include commercial activities, as well as the environment in which commerce takes place. They can have beneficial or detrimental impacts on health [16]”. While debate concerning the definition is ongoing, there is increased recognition that a diverse set of actors at the micro, meso, and macro scales are involved in the manufacture, distribution, and especially the marketing of unhealthy products [17]. As such, public health experts are calling for careful monitoring of commercial entities using the “vectors of disease” model; that is, corporations and other commercial entities in the business of unhealthy products should be approached with the same level of vigilance typically afforded to infectious disease vectors [18]. However, even in academic discourse, corporations are often regarded as faceless non-human entities, a depiction that obscures the human traits and characteristics that influence the behaviors that are at odds with public health. Yet, these powerful actors are capable of acting as vectors, ultimately causing harm at large social scales [19].

Consider the ways in which the COVID-19 pandemic has erased the rigid demarcations between infectious and non-infectious diseases—obesity, type 2 diabetes, and cardiovascular disease strongly predicted hospitalization and mortality from the SARS-CoV-2 infection [20,21,22,23,24]. It is now possible to make connections between the marketing of unhealthy products, especially to marginalized and disadvantaged communities, and COVID-19 outcomes; yet instead of considering their role in COVID-19 outcomes, the purveyors of unhealthy foods and beverages used the pandemic to bolster sales [25,26,27,28,29,30]. Major US purveyors of donuts and fast food encouraged consumers to enter their establishments for free high-sugar and high-fat products in exchange for proof of vaccination [31]. McDonald’s began placing the U.S. Department of Health and Human Services’ “We Can Do This” vaccination branding on its products [32].

The media, rather than hold purveyors of unhealthy products accountable for their role in pandemic vulnerabilities and outcomes [33], celebrated their generosity in national press (US) headlines—such as applauding McDonald’s free “Thank You Meals” for COVID-19 first responders, who could enjoy a “Double Cheeseburger, six-piece Chicken McNuggets or a Filet-O-Fish, any size soft drink, tea or hot coffee, and small fries” for a limited time [34]. It was already known, weeks before the McDonald’s “Thank You Meals” press release was disseminated, that obesity, type 2 diabetes, and cardiovascular disease were highly linked to COVID-19 hospitalizations [35]. Years of research has already shown that fast-food outlets are clustered in marginalized and socioeconomically disadvantaged communities [36], that these communities are subject to targeted unhealthy food marketing [37,38,39], and that fast-food consumption is higher in these communities [40,41].

Ultra-processed foods (inclusive of fast foods) are a worthy example of the commercial determinants of ill health in action. These are foods and beverages that are typically energy-dense, high in sodium, sugar, and fat, and low in fiber [42]. They also often include multiple non-nutritive additives [43]. Numerous studies have linked ultra-processed foods to increased risk of non-communicable diseases and early mortality [44,45,46]. In the context of mental health, it is noteworthy that, despite the immediate palatability of ultra-processed foods, consumption is linked to depression, anxiety, and mental distress [47,48,49,50,51,52].

Although the relationship between mental health and dietary patterns was formerly pushed to the fringes of academic research and clinical care [53], contemporary research has provided multiple mechanistic pathways whereby foods and beverages can influence mental outlook and cognition, as referred to in detail elsewhere [54,55]. Notably, this research also extends to relationships between diet and aggressive behavior and criminality [56,57,58]. For the purposes of the present discussion, it is worth emphasizing that several studies have shown that a single McDonald’s meal (e.g., burger and small serving of fries) places an inflammatory and/or oxidative stress burden on human physiology [59,60,61]. The combo of a McDonald’s breakfast followed by a McDonald’s burger-based lunch meal (4 h later) provoked oxidative-stress-induced impairment of endothelium-dependent vasodilation even in healthy young adults [62].

What, then, is the allure of ultra-processed meals? The high palatability of these foods appears to short-circuit behavior change related to consumption, even if individuals are aware of their potentially detrimental effects. Notably, emerging research from both animal and human studies suggests that ultra-processed foods and beverages provoke tolerance and withdrawal effects—the cardinal symptoms related to addictive substances [63,64]. In addition, accumulating research shows that consumption of high-fat, high-sugar, and high-sodium ultra-processed foods is a form of self-medication to attenuate stress—although there are acute post-consumption perceived benefits of these so-called comfort foods, the long-term consumption places a physiological burden on the body [65].

The problem, which might be viewed as a major advantage to the purveyors of ultra-processed foods, is that lifestyle and nutritional medicine literacy (including any dietary connection to mental health) is poor among otherwise highly credentialed mental healthcare providers [66,67]. Despite the lethality of obesity [68], a 2023 survey of American pediatricians shows that the majority lack confidence in approaching the topic of obesity in the clinic [69]. The recently published American Academy of Pediatrics clinical guidelines for obesity has placed emphasis on pharmaceutical interventions and surgery [70]. Thus, when Burger King engaged in a distasteful 2019 marketing campaign, supposedly promoting mental health awareness by placing Whopper-Fries-and-a-drink meals in colorful mood-based boxes (so-called Real Meals) [71], the corporation was not queried on its role in potentially contributing to depression, anxiety, and other mental disorders. Instead, top-level media ran promotional headlines such as “Burger King’s ‘Real Meals’ are about more than trolling McDonald’s. They’re about mental health” [72].

## 3. Corporate Greed as a Commercial Determinant

Entering the words “corporate greed” into Google Scholar reveals over 19,000 results, about half published in the last decade. Among other things, these references point to price gauging from pharmaceutical companies [73], shady (profit-driven) marketing practices pushing an opioid epidemic [74], manipulation of emissions tests by automobile manufacturers [75], financial industry maneuvers leading to the global financial crisis [76], and corner-cutting and dismissing safety concerns in multiple corporate sectors—from the manufacturing of aircraft to garments [77]. In a highly accessed January 2023 viewpoint paper in the *Journal of the American Medical Association*, “greed” was described as an existential threat to healthcare: “Greed harms the cultures of compassion and professionalism that are bedrock to healing care… the cycle is vicious: unchecked greed concentrates wealth, wealth concentrates political power, and political power blocks constraints on greed” [78].

The connections between corporate greed and compromised individual, community, and public health are often referred to in academic and policy discussions. However, greed as a psychological construct is missing from much of this discourse. More specifically, the ways in which individual differences in personality and specific situations can mix, as if in an enzymatic reaction, to increase greedy behavior [79]. In other words, although greed is often mentioned, it is at an abstract level, disconnected from the idea that corporate greed involves individuals and small groups. While it is understood that the legal notion that corporations are “persons” is problematic for public health [80], it is also important to remember that the persons within corporations and other commercial entities, especially those at the top of organizations, have worldviews that impact human health at scale. To understand the ways in which corporate (or more broadly, commercial) practices compromise human health at scales of person, place, and planet, we need to consider the ultimate upstream driver of the commercial determinants of health—corporate greed, which is to say, the biopsychosocial underpinnings of human greed at the state (emotional) and trait (personality) levels.

Although contemporary research on the commercial determinants of health has focused on the methods by which unhealthy products are disseminated, including marketing efforts and power-based policy manipulations [81,82], the field has paid little attention to the ways in which individual psychological traits apply at the group level and the ways in which biological and psychological vulnerabilities of consumers make them easy marks for targeted marketing. The absence of the word “greed” in the newly published Oxford University Press textbook *The Commercial Determinants of Health*, an almost 400-page text about unhealthy commercial activities [15], is emblematic of the void in the field. The same textbook does not include input from any experts in the realm of psychology, and there is little more than a few passing references to psychology. References to emotion, empathy, entitlement, and narcissism (and their variants) are nowhere to be found. These concepts are also absent in the otherwise excellent foundational paper “Defining Priorities for Action and Research on the Commercial Determinants of Health” published in the *American Journal of Public Health* [83].

## 4. Dispositional Greed

While greed has been part of discourse within theology and philosophy for centuries, there has been more academic attention to the psychology of individual differences in recent years. The etymological roots from the Gothic word gredus, meaning hunger [84], fit with modern academic definitions of “an insatiable desire for more resources, money or other” [79] and the “desire to get more at all costs, including the excessive striving for desired goods and willingness to accept that such striving may be at the expense of others” [85]. The latter adds the dimension that greed is often accompanied by collateral harm, whether intentional or not.

Several scales to measure dispositional greed have been validated in recent years. These include items such as “I always want more”, “I can never have too much money”, and “It doesn’t matter how much I have, I’m never completely satisfied” [86]. Some greed scales or subscales add additional questions related to ethics and power such as “When I play on my own, I sometimes cheat a little” [85] and “I do not enjoy sharing positions of power” [87]. These questions are important because dispositional greed has been increasingly linked to “dark triad” personality features of Machiavellianism, narcissism, and psychopathy [87,88]. Machiavellianism describes detachment from conventional morality while deceiving and manipulating others (i.e., the ends justify the means); narcissism describes elevated egocentrism, grandiosity, and desire for attention; and psychopathy describes antisocial, impulsive, and callous behavior—and all of these dark triad personality features are characterized by low empathy [67,89]. Indeed, dispositional greed is associated with diminished empathic concern and moral reasoning and increased antagonism, aggression, entitlement, willingness to make backhanded deals, and a greater ease in the justification of unethical acts [86,90,91,92,93,94].

While greed is related to envy and materialism, it is also a distinct construct [86]. In an experimental setting, if an individual outperforms their counterparts on a particular task, provoking a sense of entitlement on resource distribution, there is an even higher propensity to act greedily—twice as many people act in a greedy manner once entitlement enters the mix [95].

Although dispositional greed can be found in persons in all walks of life, in the realm of occupations, greed is not found at random. Research shows that dispositional greed clusters in banking, insurance, real estate, and extractive industries; on the other hand, dispositional greed is lower among persons in health care, education, and civil service [96]. Business students report a higher love for money than psychology students [97] and are more likely to score higher on dark triad characteristics [98,99].

The extent to which the occupational choice-dispositional greed measures are based on self-selection and early-life experiences is unknown. Although there is evidence that childhood wealth is associated with later-life greed [100,101], it has also been shown that economics education actually enhances greedy behavior among MBA students [102]. In the meantime, such occupational clustering is important in the framework of the commercial determinants of health because it invites query on the laws of attraction, which is to say, group-level greed. Research over the last several decades has supported the attraction–selection–attrition framework in which organizations are not really “faceless’”—they are populated with individuals sharing similar personalities and values [103].

## 5. Greed in Groups and Networks

“*When it comes to competition—well, if they were drowning, I’d put a hose in their mouth. I have a feeling about competitors that I’ve tried to get across at McDonald’s. Competitors are someone you learn to hate. There’s no nice way of being in business and loving your competitors.*”

—Ray A. Kroc, 1974 [104].

In addition to developing and disseminating his own myth of greatness, and the idea that he was the founder of McDonald’s (the McDonald brothers already had multiple franchises of the golden arches before the Kroc purchase in 1961 [105]), it has been said that one of Kroc’s strongest assets was a collective of top lieutenants [106,107]. Since those with higher dark triad traits are drawn to others with the same traits [108,109], and are less likely to find the signals of grandiosity and diminished empathy as repulsive [110], the study of dispositional greed warrants expansion in a collective setting. The trait-based features of the dark triad—Machiavellianism, narcissism, and psychopathy—have each been observed in collectives [111,112,113].

The concept of collective narcissism, which describes a belief in the greatness of a particular in-group, provides an example of how trait-based individual narcissism can apply to the intergroup level. For example, at the individual level, narcissism is often associated with the superficial trappings of virtue, and at larger scales, this can be observed with collective narcissism, such as support for superficial greenwashing efforts as an image-enhancing strategy rather than deeper-level pro-environmental behaviors that require deep levels of commitment [114]. Just as individual narcissism is associated with the construction of historical accounts and biographies promoting personal greatness [89], collective narcissism is also associated with the production of narratives that emphasize the greatness of the in-group and the importance of its role in history [115].

In the present context, it is important to note that dark triad features at the group scale (e.g., the resentment and hostility toward out-groups) are especially notable when the targeted out-group is considered to be a competitor, or an enemy [111]. Moreover, the targeted out-group in the context of collective narcissism, greed, Machiavellianism, and psychopathy is a “flexible” entity. Although Kroc might have been willing to, in his own words, put a hose in a drowning fast-food competitor’s mouth, he would be willing to band together with them in the face of external threat to the overall fast-food industry. In other words, the targeted out-group can easily slide from another fast-food purveyor to nutritionists, public health advocates, and academic professionals. Consider Kroc’s statement in 1975: “*What do all those nutritionists and college professors and those* [Ralph] *Nader types know? How many jobs have they ever created?*” [116].

Collective dark triad features and greed can be observed in the operations of nutritional front groups that operate in Machiavellian ways to place “industry approved” experts before the press and media while at the same time obfuscating industry’s powerful role in shaping nutritional discourse and public opinion [117]. In sourcing experts, the media has had a well-documented struggle in identifying corporate shills and the “science-y” front groups they represent [118]. The greed of leadership within professional nutritional associations—accepting funding and allowing grossly conflicting power-based relationships—can lead to corporate capture and resultant pro-industry policy positions [119,120]. It is salient that these conflicting relationships often involve individuals and small teams at the upper echelon of organizations, a place where individual traits can coalesce. Although the specific tactics of the unhealthy product industries have been elegantly critiqued by public health experts [121,122,123,124], the connection to the deeper roots of psychology and marketing (the latter increasingly inseparable from the former [71]) is rarely discussed. For example, discussions of the interplay between select scientific experts purporting the “benefits of sugar” (while minimizing any health risk), and a tight front group of sugar industry members, have focused on potential bias and conflicts of interest [125,126]. What about the intersection of dispositional greed and dark triad traits among these groups?

Working from trait activation theory, which suggests that the degree to which traits are expressed is a product of environment-based motivation [127], evidence suggests that collective moral judgement can be influenced by dark triad traits (e.g., Machiavellianism) [128]. Organizational research shows that dark triad traits promote unethical behavior that might be viewed as favorable to the organization [129] and that highly entitled employees are more willing to engage in unethical pro-organizational behavior when their personal goals are aligned with those of their organizations [130]. Even in the absence of high-level trait Machiavellianism, marketing professionals use cognitive neutralization techniques to rationalize and reframe ethically questionable intentions and behaviors [131]. Dark triad traits at the leadership level have been shown to “trickle down” to subordinates and lead to moral disengagement and less concern for ethics [132].

The idea of a culture of greed within organizations, or that individual dispositional greed can magnify and cluster at scale, is supported by research within the financial sector. For example, deference, flattery, and strict adherence to the authority of leaders—described as upward ingratiatory behaviors—are utilized by employee subordinates who are themselves high in dispositional greed [133]. One of the potential rewards of this behavior is upward promotion. From the commercial determinants of health vantage, there is a need to understand the interplay between trait and state greed [85] and how this might be accentuated if there is a clustering of individuals with dispositional greed and the dark triad traits.

## 6. Social Dominance Orientation

In the context of the commercial determinants of health, individual and group-level attraction to hierarchies and the prestige of hierarchical social systems (known in psychology literature as social dominance orientation (SDO)) is of relevance. Higher SDO scores reflect a worldview in which the social domination of certain groups (out-groups) by a high-status group is just, or good. Not surprisingly, SDO is positively associated with belief in a just world—the idea that people get what they deserve and deserve what they get. For example, if a person is obese or in poverty, they are largely to blame for their own situation [134]. Kroc maintained neoliberal worldviews and belief in a just world: “*I have always believed that each man makes his own happiness and is responsible for his own problems*” [135].

SDO is associated with greed, ruthlessness, and a win-at-all-costs outlook [136]. Like dispositional greed, SDO is associated with cognitive mechanisms that rationalize disparities between groups and diminished awareness that power gained from the dominant social position is used for personal gains [137,138,139]. In group settings, SDO has been linked to increased justification of self-serving decision making at the expense of lower-level subordinates [140]. Kroc’s financial empire rested upon a massive workforce paid in paltry wages; Kroc and McDonald’s vehemently opposed a federal minimum wage. In 1972, Kroc personally gave Richard Nixon over USD 250,000 in his reelection bid. Kroc called it generalized “insurance in the free enterprise system” [141], but critics did not mince words about the situation: “*I think it is obscene for a man to give $255,000 to a political candidate at the same time he is fighting against paying someone $2 an hour to work in a hot kitchen*”, said Senator Harrison Williams, Chair of the Senate Labor Committee [142]. Nixon dutifully vetoed the minimum wage bill [143]. In the 1980s, Kroc’s top lieutenant, Fred L. Turner, was making 82 times more per hour than his front-line workers [144].

Belief in a just world and SDO can work in tandem to justify, albeit in slightly different ways, unequitable systems and maintenance of a literally unhealthy status quo [145,146]. To maintain a position of high status, individuals high in SDO are vigilant against any threats to status, including policies that are intended to address social inequalities. Like dispositional greed, individuals high in SDO have lower empathy [147] and choose occupations that emphasize financial wealth and are disconnected from social welfare [148].

Collectively, the research cited above can inform discussions on the tactics used by the unhealthy product industries. This can aid understanding how justifications for strategies are formed, even when there is ample prevailing evidence of harm. Kroc’s opinion that his business of fast food is akin to “showbusiness” is also a reflection that many “actors” are involved. Kroc was certainly a thought leader in this regard. Thirty years later, experts in marketing and communications published a textbook entitled *There’s No Business that’s not Show Business* by Prentice Hall, in 2004 [149]. The authors write that showbusiness is “*a well-kept secret behind the corporate curtain…used to dazzle customers, to communicate internally, to launch new products, and to attract new business partners. Show business is extremely effective. It helps to differentiate products and brands. It creates powerful connections with customers. It attracts media attention*” [149]. Through this lens, we can see how dispositional greed and justification intersect among these various actors as they put on a show for the target audience—kids.

## 7. Showbiz—Going after the Kids

“*Our move to the suburbs was a conscious effort to go for the family business. That meant going after the kids. We decided to use television, so we created our own character, Ronald McDonald*.”

—Fred L. Turner, 1973 [1].

**Figure 1:**
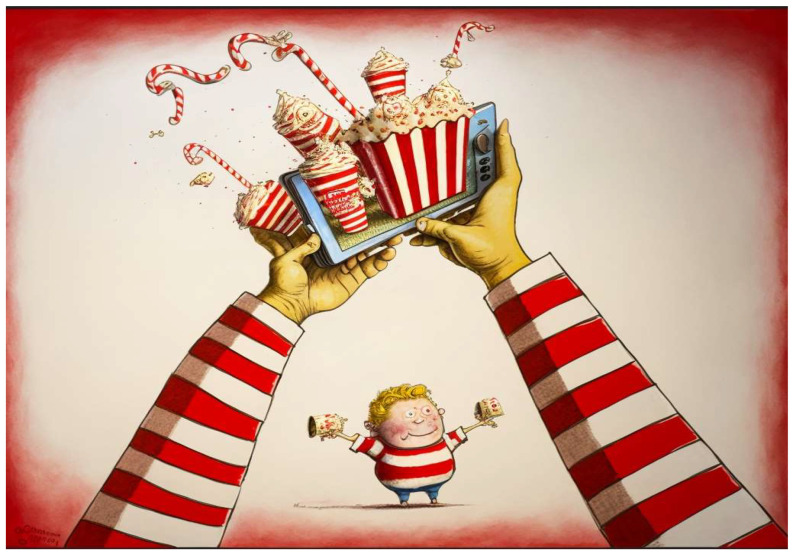
**Marketing directly to children**: Brand “characters” such as Ronald McDonald are recognized by 90% of 6-year-old children in the USA [150]. Studies show direct marketing to children causally influences child preferences for ultra-processed foods [151] (Art copyright, author S.L.P.)

Kroc’s top man, Fred L. Turner, his handpicked president, was also interviewed in the *Time* magazine cover story on Ray Kroc. Perhaps in his own unbridled enthusiasm, Turner’s comments (above) exposed the intentional marketing scheme used to magnify the sales of fast food. It is hard to imagine a contemporary president or chief executive officer within the unhealthy product industry boldly admitting their strategy was going after the kids, luring them in with a “character” (Figure 1).

Under Kroc and Turner, McDonald’s advertising was based on the braggadocio claims of feeding the masses—“Over Eight Billion Sold” was the claim in 1971, with the added subtext that if the burgers were “*laid out flat end-to-end it would circle the Earth 18 times*” [152]. Yet, faced with criticisms of nutritional inadequacies of fast food, a McDonald’s public relations official minimized implications of mass consumption in testimony to the United States Congress—“*our menu is always supplemented by foods eaten elsewhere*” [153]. During this 1979 testimony, it was revealed that McDonald’s sent 100,000 copies of its “Nutrition Action Pack” booklet to American elementary schools in 1975 and that millions of children were educated with the books. While McDonald’s did not directly commercialize any of their products, the cover of the booklet illustrates the food groups. The “dairy” group depicted a square slice of cheese (think cheeseburger), a shake, an ice cream cone, and an ice cream sundae. The burger is depicted squarely in the protein group. The preface to the booklet was written by Harvard nutritionist Fredrick Stare, an agribusiness consultant who vigorously defended the sugar and fast-food industries [123,154,155,156]. McDonald’s followed up with consumer literature emphasizing Kroc’s view on personal responsibility—“*Good nutrition is up to you… as you select items from our menu, consider their nutrient content and the part they will play in your whole day’s nutritional intake. Adjust your other meals accordingly*” [157].

In the 1980s, the corporation also took out full-page ads in dozens of national magazines (e.g., *Working Mother* magazine) to inform consumers about their wholesome fare: Accompanied by three simple images of a large bottle of milk, a large unpeeled potato, and a paper container of raw ground beef, all balanced vertically on each other, the ad read “*What We’re All About. Meat and Potatoes. Milk and Bread. Good, basic nutritious food. Food that’s been the foundation of well-balanced diets for generations*” [158]. Kroc’s message to his critics, the idea that he was simply following nutritional tradition dating back to Neolithic times, echoed onto the pages of print media. While the notion of Machiavellian marketing—the behind-the-scenes lobbying efforts of commercial actors on policy—has been well-described [159], the extent to which dark triad features aid in the justification of advertising junk food to children and vulnerable communities remains unexplored. Critics have pointed out that creatives in the field of advertising and public relations have carefully concealed the ways in which learnings from consumer psychology, and their own observations, are leveraged to produce propaganda [160]. It would seem to take a significant amount of strategic ignorance (i.e., willful avoidance of evidence concerning negative social impacts of decision making [161]) to craft an ad campaign whereby readers of *Working Mother* are informed that Kroc is running what amounts to a global chain of health food stores.

Decades before “experiential marketing” entered the lexicon of communications and marketing education (i.e., the idea that consumers are rational and emotional human beings concerned with achieving pleasurable experiences) [162], McDonald’s understood the value of experience. In the 1970s, a senior McDonald’s public relations executive captured the strategy: “*We offer people more than just fast-food. It’s an experience. It’s an experience of fun, folks, and food. We’ve sold 18 billion hamburgers, but we’ve sold them one at a time*” [163]. Part of the “going after the kids” vis-à-vis “experience” was the careful tailoring of the character described by Turner—that is, Ronald McDonald [164]. Purveyors of unhealthy products soon realized they needed their own characters to aid in delivering the experiential framework, and before long, Joe Camel was delivered by the tobacco product manufacturer, RJ Reynolds. Joe Camel was first crafted in Europe in 1974, and internal tobacco industry documents show the calculated effort behind the character and how it reaches “about as young as you can get, and aims right at the young adult smoker Camel needs to attract”; further memos show that when test-marketed in the US in 1984-85, it was a “booming success” [165]. The group-level traits and degree of strategic ignorance that facilitated such marketing campaigns are unknown. What is known is that by the 1990s, Ronald McDonald, the Golden Arches, and Joe Camel received close to 90% recognition by 6-year-old US children [150], just shy of the recognizability of Santa Claus [166].

While some in public health and psychology drew formal parallels between Ronald McDonald and Joe Camel [167], the subject was discussed in detail by legal scholars [168,169]. John Banzhaf, the Dr. William Cahan Distinguished Professor at George Washington University Law School, said that “*Sending Ronald McDonald into schools to serve as a ‘Health Ambassador’ teaching children about fitness is as subversive as using Joe Camel to teach kids about proper breathing techniques to help counteract the effects of smoking*” [170]. McDonald’s CEO James Skinner claimed that “Ronald has never sold food to kids in the history of his existence” [171], illustrating the ease with which justifications are made. Research clearly shows that even very young children recognize the links between Ronald McDonald and fast food and that such recognition is associated with favorable views of the food [172]. Volumes of research also show that exposure to McDonald’s advertising and promo gimmicks is associated with unhealthy food preferences and increased fast-food consumption [173,174,175]. More specifically, we know that McDonald’s ads targeted to children increase the odds that parents who would not ordinarily consume fast food will follow the lead of the child’s desire [176]—which is precisely the strategy Fred L. Turner set up when he was “going after the kids”.

In the 1970s, McDonald’s expanded its fare into public school lunches. In Benton, Arkansas, the high school switched from a traditional school lunch to McDonald’s burgers, fries, and hot dessert pies [177]. The McDonald’s move into schools was given the full support of Dr. George Graham, who told Newsweek that “*you can hardly beat a Big Mac for an excellent concentration of high-quality protein, and if you add to that the vitamins and minerals from the French fries and the milk, you have a good supply of vitamins A, B, and C as well. McDonald’s maintains superb quality control. Perhaps most important is the fact that kids eat their food*” [177]. Dr. Graham, a Johns Hopkins University nutrition professor, was an idealogue who argued that individuals were responsible for their own states of poverty; he was a consistent defender of large corporations and agri-businesses [178].

McDonald’s entry into schools, both directly and indirectly through its branding and entertainment, has been highly successful. In a study involving children aged 3 to 5, recruited from preschools in low-income communities, it was shown that children preferred the tastes of foods and drinks if they thought they were from McDonald’s (i.e., presented in McDonald’s wrapping); this effect was pronounced in children who ate more frequently at McDonald’s than in children from homes with more television sets [179].

This sort of showbiz endures. It is not by chance that the McDonald’s Corporation spent over USD 770 million on advertising in 2019 [6]. Much of that advertising is directed at children [180]. What remains unclear is the extent to which adults and adolescents are mindful (or completely unaware) that they can be entertained into states of chronic disease and lowered mental outlook. To what extent are adults and adolescents aware that four industries (tobacco, unhealthy food, fossil fuel, and alcohol) are responsible for at least a third of global deaths per year [181]? Available research, described below, suggests that awareness of the manipulative and profit-driven tactics of the unhealthy product industries is an important part of the solution.

## 8. Solutions—Turning the Corner

Academic exploration within the context of commercial determinants of health has emphasized a need for greater awareness of the tactics used by purveyors of unhealthy products. Many of these tactics cluster around the spread of propaganda in advertising and corporate public relations. Without proper media literacy training, young people are especially vulnerable to the effects of propaganda and advertising as manipulative devices on their behavior [182]. On the other hand, when adolescents are made aware of the manipulative and unfair practices of the food industry, research shows they view junk food marketing in a less favorable light and are motivated to eat in healthier ways [183,184]. When adults are presented with clear messaging highlighting the ways in which food is engineered on three fronts—physiological (via sugar/fat/sodium/additives), cognitive (advertising/marketing), and environmental (e.g., product shelf placement)—they are more likely to support policy reform [185].

These findings illustrate that increased awareness is an important prerequisite in tackling the commercial determinants of ill health. Along those lines, it is interesting to note that trait mindfulness is negatively correlated with dispositional greed [186]. Moreover, research has linked mindful parenting to lower levels of greed among children and adolescents [187,188]. Mindful parenting describes the classic features of mindfulness—moment-to-moment awareness that is characterized by open, non-reactive, non-judgmental attention [189]—while emphasizing compassion and empathy [190]. Recall that empathy, or lack thereof, is the common thread in personality features under current discussion, including dispositional greed. Since empathy is akin to a skill that can be taught [191], it suggests that late childhood and early adolescent interventions addressing cognitive empathy can provide an important part of moral and social education [192,193]. By extension, it could be argued that awareness of the potential harms of greed and cognitive empathy deficits should be incorporated into related education, including media literacy.

The available research also indicates the need for a deeper understanding of the developmental origins of greed and its correlates. Core beliefs—the mental representations individuals hold about themselves, others, and the world in general—appear to be instrumental in the early-life origins of greed; the unbridled quest for money, material goods, and/or power that otherwise characterizes individuals with high dispositional greed may be a compensatory behavior resulting from insecurity and other negative core beliefs [194]. Until recently, the field of personality psychology was mostly descriptive; advances are allowing for a deeper understanding of the developmental origins of individual differences (including underlying mechanisms and environment/socialization interactions) and the behaviors that have the potential to impact society writ large [195,196]. The field of consumer psychology has made important strides in understanding the motivations that drive consumer choices [197]; future work might examine the intersections of the motivations and tactics used by the unhealthy product industry and individual or community vulnerabilities to such messaging and tactics.

Moving the research forward will require modeling of these interrelated phenomena in order that they might be studies with specificity, especially in the context of the commercial determinants of health. Based on the framework we are presenting here, dispositional greed at the individual level has the potential to coalesce at the group level. This is testable and reflects similar models that have been subject to robust international research—the harmful effects of the dark triad of personality have been the subject of much research in business management, especially in the way that these personality features manifest in toxic leadership within organizations [198,199]. The consequences for the wellbeing of organizational subordinates have been well-described in the literature [200]. According to our framework, the inclusion of dispositional greed should provide similar (negative) observations on wellbeing, beyond organizations per se. For example, we would expect to see higher levels of justification for the marketing tactics used by business personnel (to push unhealthy product sales), especially those who score higher on dark triad traits and dispositional greed. Based on our framework, an ad that suggests McDonald’s is in the business of meat, potatoes, bread, and milk would appear justifiable in this group. As described above, public awareness of the manipulative profit-driven tactics of the unhealthy product industry enhances support for policy changes and personal shifts toward healthy behavior. We suggest that this will be enhanced with education and awareness of the ways in which dispositional greed can manifest in those manipulative tactics.

Part of the solution will be a closer academic examination of the ethical ramifications of marketing unhealthy products in the Anthropocene. Fast-food corporations are particularly skilled at inserting their greed-driven agendas into cultural critiques, offering messages that allow people to feel good about their choices (whether or not those choices are good for personal, public, or planetary health) [201]. Fast-food companies have not convincingly met the five principles of the TARES Test for ethical marketing—Truthfulness (of the message), Authenticity (of the persuader), Respect (for the persuadee), Equity (of the persuasive appeal), and Social Responsibility (for the common good). [202]. The future of global health and equity will rely on the commercial sector and thus a framework that considers ethics in compliance mechanisms for commercial entities [203].

More significantly, in the wider context, this discussion feeds into the fundamental value systems underpinning the social, environmental, and spiritual challenges of the Anthropocene, which has been characterized by human greed and more extractive mindsets [89,204]. Large-scale population trends of increasing narcissism and declining empathy [205,206] are mirrored in attitudes to the environment and to others, including decreased empathy for marginalized groups [207]. Again, these mindsets have early-life origins and mirror cultural values and priorities [208]. This calls for a deeper cultural change—one that supports the health of people, places, and planet, rather than undermines it [204]. The fact that these collective attitudes and personality traits have changed over time and reflect social environmental conditions offers hope that more evolved and compassionate approaches to life are possible [209].

## 9. Conclusions

“*All too often in the choice between the physical health of consumers and the financial wellbeing of business, concealment is chosen over disclosure, sales over safety, and money over morality. Who are these persons who knowingly and secretly decide to put the buying public at risk solely for the purpose of making profits, and who believe that illness and death of consumers is an apparent cost of their own prosperity?*”

—Federal Judge H. Lee Sarokin. 1992 [210].

In this assessment by Judge Sarokin, taken from the case of Susan Haines vs. leading tobacco companies, he asks “who are these persons?”, referencing the otherwise obscured individuals who sit behind the upper echelons of the unhealthy product industries. It is a question that should be of high-level interest to those engaged in commercial determinants of health research and discourse. It is not enough to know their tactics. There is a need to know more about their traits and, in particular, the environments in which state and trait greed are drawn out. There is a need to understand the ways in which dark traits coalesce in commercial groups and intersect with the dispositional greed of policymakers who make no regulatory policy. There is a need to explore the ways in which these traits justify the maintenance of an unhealthy status quo.

Public health professionals have long since urged a greater focus on the “causes of the causes”—that is, rather than look exclusively at downstream factors such as neighborhood walkability, look upstream to income, wealth, education, and structural institutions, as the fundamental causes of wide-ranging health outcomes [211,212]. Examining the commercial determinants of health is a logical part of this work. However, it is our contention that the construct, at least so far, has not ventured far enough upstream, to the points of origin. It might be better to consider the causes of the “causes of the causes”—there you will find dispositional greed, social dominance orientation, features of the dark triad, and system-justifying beliefs permeating the wellspring, leading to toxic waters that flow downhill into an unjust global system. The available evidence tells us what will be sparse at the wellspring—empathy.

In the wider sense, the commercial determinants of health reflect attitudes, behaviors, and worldviews that underpin the mounting challenges to human flourishing and environmental sustainability in the Anthropocene. From this vantage, the greatest threats to health are not “technical” challenges but social, relational, and “attitudinal” challenges, which will not be solved without addressing the value systems that created our grand challenges in the first place. This is the basis for calls for a cultural shift that places greater value on kindness, generosity, reciprocity, and mutualistic values in seeking the greatest common good [204]. Yet, mutualistic worldviews, by their nature, cannot be forced. They are best cultivated from the roots early in life with the understanding that deeper purpose and meaningful relationships with the world upon which we depend “*thrive on mutual respect, reciprocity, kindness, and yes that four-letter word… love*” [213].

## Data Availability

Not applicable.
